# Exploring the Discrepancies in the Biological Activities of Extracts From *Guadua angustifolia* Var. Bicolor Londoño Collected in Two Different Sites

**DOI:** 10.1002/cbdv.202500174

**Published:** 2025-05-03

**Authors:** João Kazlauckas, Maria Tereza Grombone Guaratini, Paulo Roberto H. Moreno

**Affiliations:** ^1^ Departamento de Química Fundamental Instituto de Química, Universidade de São Paulo Sao Paulo Brazil; ^2^ Instituto de Pesquisas Ambientais, Núcleo de Uso Sustentável da Biodiversidade Sao Paulo Brazil

**Keywords:** antioxidant, anti‐tyrosinase, bamboo, *Guadua angustifolia* var. bicolor Lodoño, phytochemical composition

## Abstract

*Guadua angustifolia* var. bicolor Lodoño is a Colombian bamboo species that has shown promising medicinal properties. This study aims to investigate the differences in the chemical composition and biological activity of extracts from two different populations of *G. angustifolia* var. bicolor, collected in Bauru and Tatuí, Brazil. The extracts were analyzed using high‐performance liquid chromatography‐tandem mass spectrometry, and their anti‐tyrosinase and antioxidant activities were evaluated using the enzyme tyrosinase and diphenyl‐1‐picrylhydrazyl, respectively. The crude extract was prepared using a Soxhlet apparatus, followed by a solid‐liquid extraction and, finally, a column chromatography. The results showed that the subfractions from the two populations had different chemical compositions, which may explain their different biological activities. The Bauru extracts contained higher levels of phenolic compounds and fatty acids, which are known for their antioxidant and anti‐tyrosinase activities. These extracts were also found to be the most active samples. These findings suggest that the chemical composition of bamboo extracts can vary depending on the origin of the sample.

## Graphical abstract

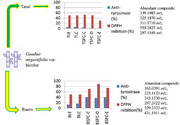

## 1 Introduction

Bamboos are angiosperms of the order Poales, belonging to the Poaceae family, subfamily Bambusoideae. The expected classes of secondary metabolites found in this subfamily include phenolic acids, flavonoids, coumarins, and tannins [1, 2, 3]. It is possible to state that these plant species are capable of adapting to different habitats, as they are easily found in forests around the world, except on the European and Antarctic continents [1, 3, 4].

**FIGURE 1:**
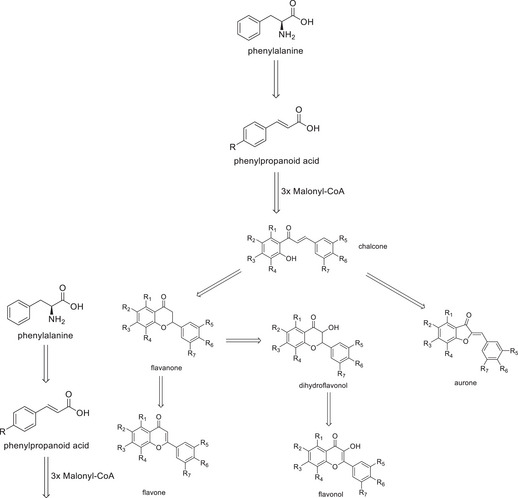
Flavonoid biosynthetic pathway summarized. R_1_ = R_3_ = OH, OCH_3_, O^‐^ Glycoside, Glycoside; R_2_ ─ R_4_ ═ R_5_ ═ R_6_ ═ R_7_ ═ H, OH, OCH_3_.

Among the above‐mentioned substance classes, the main ones found in bamboo species are phenolic acids and flavonoids. This abundance can be associated with the fact that both are directly related to the same biosynthetic pathway [5]. In summary, the pathway (Figure 1) begins with the amino acid phenylalanine, which is converted into a phenylpropanoid acid and then gives rise to a chalcone, which can finally be used to generate more specific structures of flavonoids, such as aurones, flavanones, flavones, dihydroflavonols, flavonols, and their respective *C‐*­ and *O‐*glycosides.

In Asian culture, bamboo is a traditional plant of significant importance, utilized for both construction and medicinal purposes since ancient times. Numerous studies in the literature highlight the medicinal applications (anti‐inflammatory, antimicrobial, antioxidant, antitumor, among others) of bamboo extracts and correlate them with the potential molecules responsible for these biological activities, which range from essential oils to benzoic acid derivatives and flavonoids [1, 3, 4].

The potential biological applications of extracts derived from American bamboo species have been the subject of recent research, although this area of study remains in its early stages. To assess the potential applications of extracts from American bamboo species, various bioactivity assays were conducted. *Guadua angustifolia* Kunth [6, 7], *Guadua chacoensis* (Rojas Acosta) Londoño & P. M. Peterson [6, 8], and *Merostachys pluriflora* Munro ex E. G. Camus [9] extracts exhibited antioxidant and anti‐tyrosinase activity. In addition to antioxidant properties, *Aulonemia aristulata* (Döll) McClure, *Chusquea meyeriana* Rupr. ex Döll Apoclada simplex McClure & L. B. Sm., and *Merostachys riedeliana* Rupr. ex Döll extracts demonstrated antimicrobial and photoprotective activities [6, 10].

**FIGURE 2:**
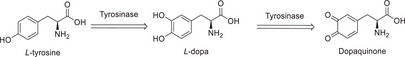
Steps in melanin synthesis in which tyrosinase participates.

Among these properties, the potential inhibition of the enzyme tyrosinase (EC 1.14.18.1), involved in the first steps in melanin biosynthesis, which confers pigmentation to human skin, is particularly noteworthy [11, 12, 13]. In this pathway, the enzyme acts in the initial stages, converting *L*‐tyrosine into 3,4‐dihydroxy‐*L*‐phenylalanine (*L*‐Dopa), and then into dopaquinone (Figure 2) [11]. Several compounds found in bamboo, such as luteolin, rutin, and apigenin, have been documented in the literature as tyrosinase inhibitors [11, 12, 13].

This research focused on the species *G. angustifolia* var. bicolor Londoño, first identified in Colombia, which is present in several South American countries [12, 13]. American bamboo species are rarely discussed in the literature, despite their immense diversity and potential for human use. In the last years, some studies were published aiming to better understand their phytochemical composition and their use in human health [2, 4].

The present study aims to investigate the potential factors contributing to the observed discrepancy in the biological activity of *G. angustifolia* var. bicolor extracts obtained from specimens collected in Bauru and Tatuí, both located in São Paulo, Brazil.

## 2 Results and Discussion

During the collection of foliar samples, visual differences were observed. The Bauru specimens displayed a chlorotic, desiccated appearance, indicating poor health. In stark contrast, the Tatuí specimens exhibited a vibrant, green coloration, reflecting their healthy condition.

The antioxidant activity (Table 1) revealed a 10‐fold higher IC_50_ value for Tatui Leaf Extract (TLE) (1700 µg/mL) compared to Bauru Leaf Extract (BLE) (163.55 µg/mL). As the diphenyl‐1‐picrylhydrazyl (DPPH) assay works mainly by hydrogen transfer, phenolic compounds are the main agents for the activity measured [14]. The observed disparity in IC_50_ values suggests a difference in the phenolic and flavonoid concentration between the two extracts. In the tyrosinase inhibition assays, the discrepancies observed were more intense between the extracts, at the tested concentrations. BLE exhibited a tyrosinase inhibitory activity of 12.86%, while TLE demonstrated a much lower activity of 4.57%, approximately threefold lower, both at the same concentration (700 µg/mL).

**TABLE 1:** Anti‐tyrosinase and antioxidant activity from extracts.

Sample^a^	Sample in anti‐tyrosinase (µg/mL)	Tyrosinase inhibition (%)	IC_50_ (µg/mL) antioxidant activity^b^
BLE	700	12.86 ± 2.17	163.55^A^ ± 8.64
TLE	700	4.57 ± 1.30	1700^B^ ± 0.99
^c^Kojic acid	5.2	50 ± 0.70	—
^d^Quercetin	—	—	4.1^C^ ± 0.27

^a^ Bauru Leaf Extract (BLE), Tatui Leaf Extract (TLE), ^b^Concentration required to inhibit 50% of the oxidizing potential (IC_50_), ^c^positive control tyrosinase inhibition, and ^d^positive control antioxidant. The results presented are the weighted average of the tests performed (*n* = 3), together with their respective standard deviation. Different capital letters in the table indicate statistically significant differences between the means. According to ANOVA and Tukey's post‐test (*p* < 0.05), there is a statistically significant difference in quercetin between each sample compared to the Student's t‐test.

A solid‐liquid extraction fractionation was performed using chloroform because in previous studies this was the most active fraction in bamboo extracts [15]. This process generated fractions from Bauru (BLC) and Tatui (TLC). Like the crude extracts, the fractions exhibited differences in biological activity (Table 2). BLC exhibited superior tyrosinase inhibitory activity, achieving 16.46% inhibition at a concentration of 400 µg/mL, with an IC_50_ value of 482.14 µg/mL. In contrast, TLC demonstrated significantly lower inhibitory activity, with 5.08% inhibition at 500 µg/mL and an IC_50_ value of 623.75 µg/mL. Based on prior research, these results suggest that phenolic and flavonoid compounds, most likely in their aglycone forms, are present in the chloroform fractions [15] and contribute to the observed activity [3, 12, 13].

**TABLE 2:** Anti‐tyrosinase and antioxidant activity from chloroform fractions.

Sample^a^	Sample in anti‐tyrosinase (µg/mL)	Tyrosinase inhibition (%)	IC_50_ (µg/mL) antioxidant activity^b^
BLC	400	16,46 ± 2.45	482.14^A^ ± 8.45
TLC	500	5.08 ± 0.67	623.75^B^ ± 5.55
^c^Kojic acid	5.7	59 ± 0.90	—
^d^Quercetin	—	—	4.3^C^ ± 0.87

^a^ Bauru Leaf Chroloform (BLC) and Tatui Leaf Chroloform (TLC).^b^Concentration required to inhibit 50% of the oxidizing potential (IC_50_). ^c^positive control tyrosinase inhibition and ^d^positive control antioxidant. The results presented are the weighted average of the tests performed (*n* = 3), together with their respective standard deviation. Different capital letters in the table indicate statistically significant differences between the means. According to ANOVA and Tukey's post‐test (*p* < 0.05), there is a statistically significant difference in quercetin between each sample compared to the Student's t‐test.

Subsequently, the constituents of the active fractions were separated by silica gel column chromatography, yielding five subfractions (SFC A–E) for each specimen. The bioactivity results showed remarkable differences among the subfractions (Table 3). In the tyrosinase inhibition assay, the samples BSFC‐C (Bauru Subfraction Chloroform‐C), BSFC‐D (Bauru Subfraction Chloroform‐D), and BSFC‐E (Bauru Subfraction Chloroform‐E) displayed high inhibition percentages: 40.66% at 262 µg/mL, 37.14% at 273 µg/mL, and 39.78% at 225 µg/mL, respectively. On the other hand, the corresponding Tatui subfractions exhibited considerably lower inhibitory activity, ranging from 0.45% to 3.42% at similar concentrations. Regarding antioxidant activity, the BSFC‐D sample at 675 µg/mL stood out with the highest DPPH inhibition potential (88.62%), followed by BSFC‐E (74.58%) at 387 µg/mL, and BSFC‐C (70.62%) at 650 µg/mL. Tatui Subfraction Chloroform (TSFC) samples also exhibited antioxidant activity but with a lower potential as free radical scavengers at similar or larger concentrations.

**TABLE 3:** Anti‐tyrosinase and antioxidant activity from chloroform subfractions.

Sample^a^	Sample in anti‐tyrosinase (µg/mL)	Tyrosinase inhibition (%)	Sample in antioxidant (µg/mL)	DPPH inhibition (%)
BSFC‐C	262	40.66 ± 3.49	650	70.62 ± 0.75
TSFC‐C	350	3.42 ± 2.22	864	52.66 ± 1.23
BSFC‐D	273	37.14 ± 1.06	675	88.62 ± 3.89
TSFC‐D	323	1.06 ± 1.05	753	50.44 ± 5.12
BSFC‐E	225	39.78 ± 0.68	387	74.58 ± 1.99
TSFC‐E	260	0.45 ± 0.5	298	30.45 ± 2.50
^c^Kojic acid	5,1	50.5 ± 0.70	—	—
^d^Quercetin	—	—	4.5	52 ± 1.33

^a^ Bauru Subfraction Chloroform‐C (BSFC‐C), Bauru Subfraction Chloroform‐D (BSFCD‐D), Bauru Subfraction Chloroform‐E (BSFC‐E), Tatui Subfraction Chloroform‐C (TSFC‐C), Tatui Subfraction Chloroform (TSFC‐D) and Tatui Subfraction Chloroform‐E (TSFC‐E), ^c^positive control tyrosinase inhibition and ^d^positive control antioxidant.

**FIGURE 3:**
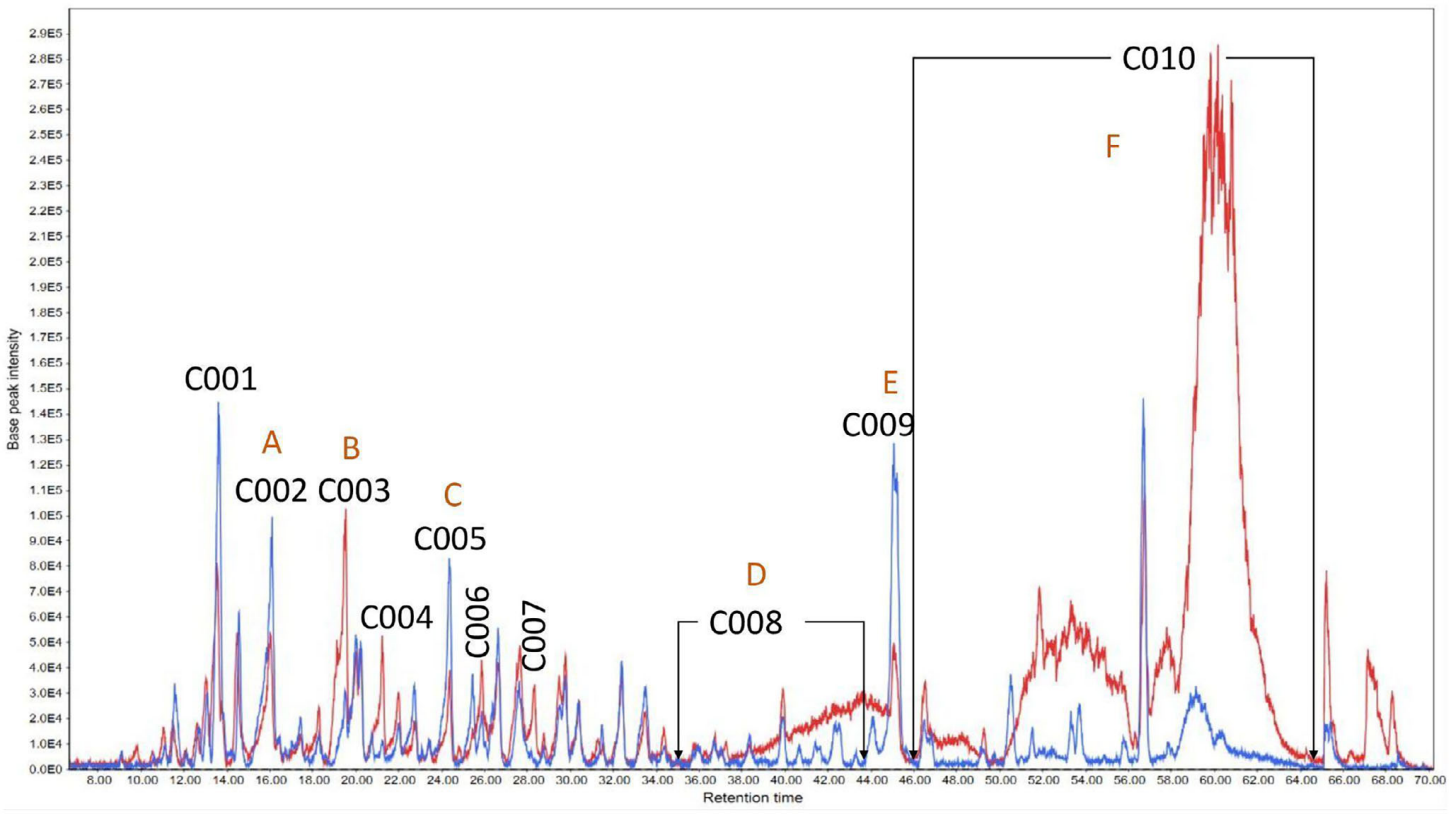
Total ion chromatogram of Bauru Subfraction Chloroform‐C (BSFC‐C) (blue) and Tatui Subfraction Chloroform‐C (TSFC‐C) (red). Deconvoluted regions: C001: 13.3–14 min; C002: 15.0–16.5 min; C003: 18.5–20 min; C004: 20.5–21.5 min; C005: 23.6–24.6 min; C006: 25.0–25.6 min; C007: 27.0–29.0; C008: 31.0–44.0 min; C009: 44.5–46.0; C010: 46.0–65.0 min. (A) p‐coumaric acid; (B) Uridine; (C) Porphobilinogen; (D) Cyclic fatty acids; (E) Acyclic terpene; (F) Cyclic fatty acid.

**FIGURE 4:**
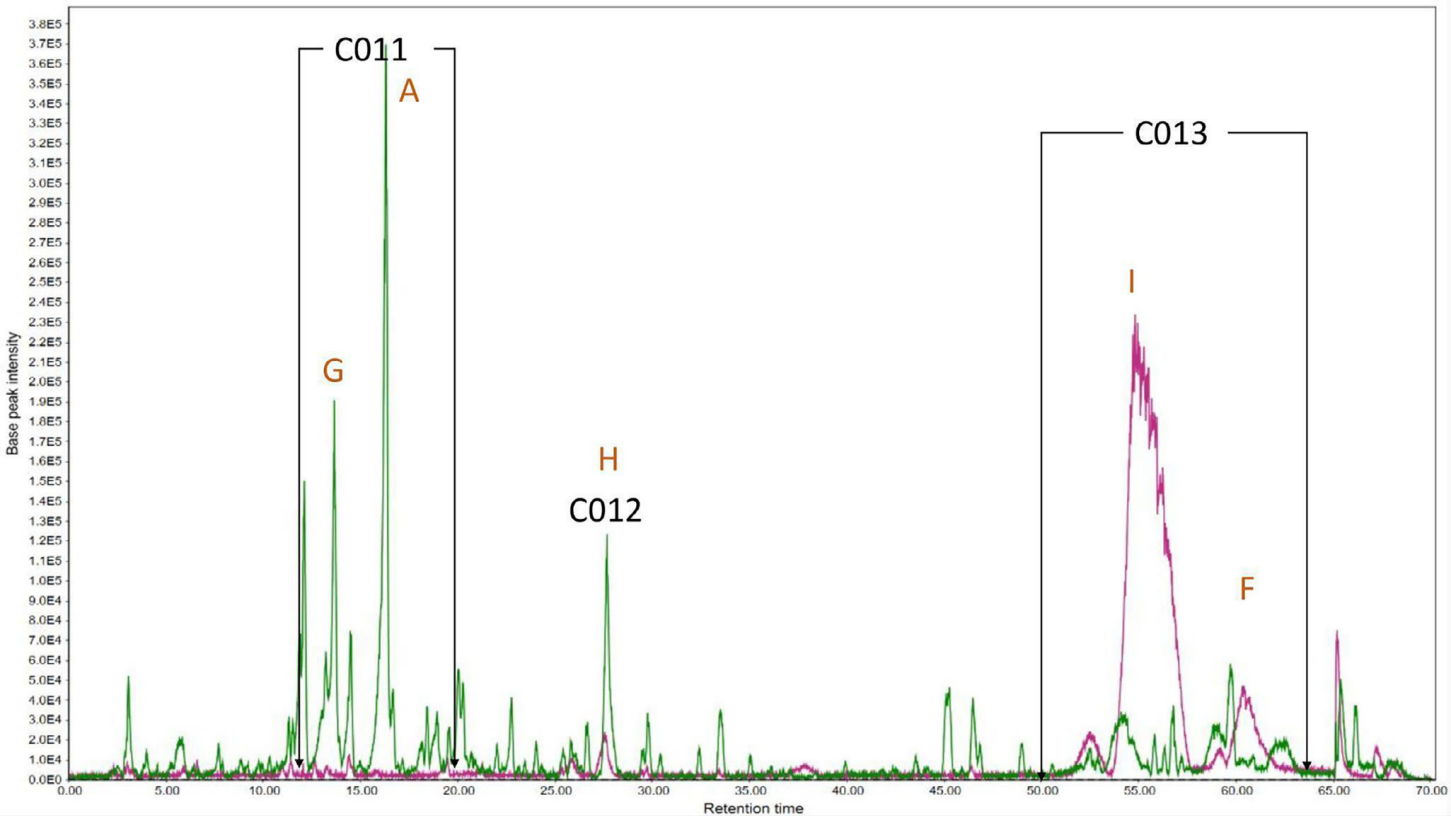
Total ion chromatogram of Bauru Subfraction Chloroform‐D (BSFCD‐D) (green) and Tatui Subfraction Chloroform (TSFC‐D) (purple). Deconvoluted regions: C011: 11.0–17.0 min; C012: 27.20–28.20 min; C013: 51.0–64.0 min. (A) p‐coumaric acid; (F) Cyclic fatty acid; (G) Protocatechuic acid derivative; (H) Hydroxylated unsaturated long chain fatty acid; I: not identified.

**FIGURE 5:**
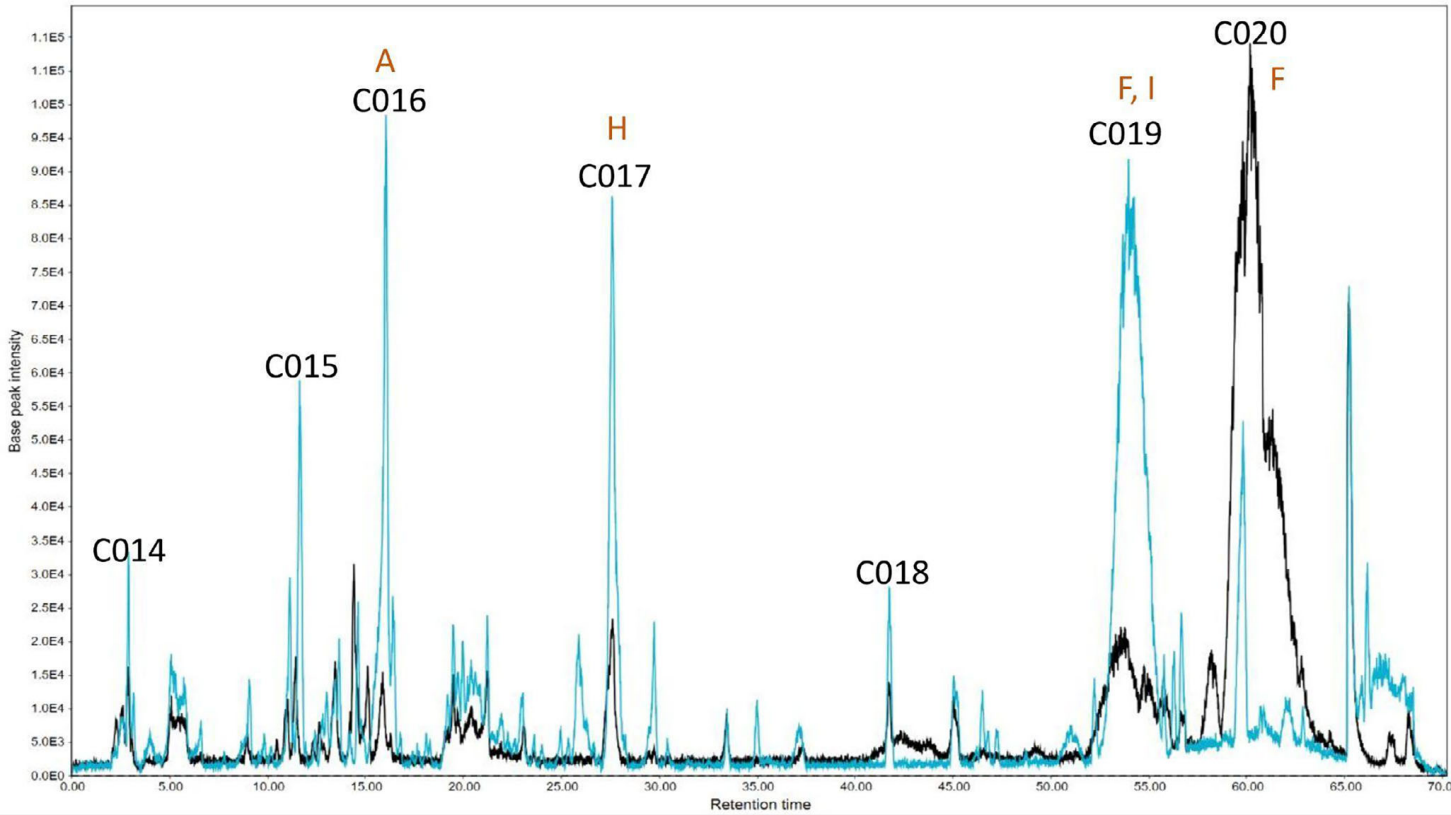
Total ion chromatogram of Bauru Subfraction Chloroform‐E (BSFC‐E) (blue) and Tatui Subfraction Chloroform‐E (TSFC‐E) (black). Deconvoluted regions: C014: 2.0–3.5 min; C015: 10.8–12.0 min; C016: 15.0–17.0 min; C017: 27.0–28.5 min; C018: 41.5–42.0 min; C019: 52.5–57.0; C020: 59.0–63. min. (A) p‐coumaric acids; (F) Ciclic fatty acid; (H) Hydroxyl unsaturated long chain fatty acid; (I) Can´t be identified.

A high‐performance liquid chromatography‐tandem mass spectrometry (HPLC‐MS/MS) analysis was conducted to determine the substances within the subfractions responsible for the observed results. The chromatograms obtained are presented (Figures 3, 4, 5) as pairs, each one comparing a subfraction from Bauru with its equivalent from Tatui. At first glance, it is evident that their compositions are visually distinct; while some peaks are the same, the major difference is in area, size, and shape. For enhanced accuracy, the chromatograms were analyzed and deconvoluted into specific regions demarcated C001‐020 as can be seen in Figures 3, 4, 5. These regions were selected based on their differences in peak height, shape, and area, serving as the main clues to investigate the divergent biological activities between the specimens.

Regions C001, C002, C003, C004, C011, C014, C015, and C016 contain more polar molecules, such as benzoic and phenylpropanoic acids, glycosylated molecules, and some short‐chain fatty acids. Aglycone flavonoids are most likely found in C005, C006, C007, C012, and C017, with the polyhydroxylated ones eluting first, followed by the methoxylated ones and steroids. Less polar molecules such as terpenoids and long‐chain fatty acids may occur in C009, C010, C013, C019, and C020.

The subfractions‐C (Figure 3) showed the largest difference in regions C008 and C010, mainly in TSFC‐C with higher areas. The deconvolution allowed us to observe pseudomolecular ions [M‐H]^‐^ 339.1985, 325.1870, and 311.1710 *m/z*, in C010, whose concentrations were extremely high in TSFC‐C. Considering the activities measured, these compounds do not have an important role in tyrosinase inhibition or DPPH scavenging. These three ions exhibited a similar fragmentation pattern with a characteristic fragment at 183.0111 *m/z*, suggesting a structural similarity among them. Each compound presented a difference of approximately 14.02 Da from the other, suggesting an extra ‐CH_2_ unit. The MS Finder database suggested canrenone (340.2154 Da) as a potential candidate for the ion at 339.2030 *m/z*, with a corresponding mass spectrum available in the MassBank of North America [16]. However, this is not a natural compound but rather a well‐known active metabolite from the drug spironolactone [17], which rules out this suggestion. Nonetheless, MS‐Finder suggested compound class options fatty acids or terpenoids. Additional literature search gives more support for cyclic fatty acids since the main characteristic for this class is to present only two fragment ions on MS/MS [18], whether terpenoids normally display more fragments [19].

On the other hand, regions C001‐C007 were visually too similar with small differences in peak height. Thus, based on the differences presented between the chromatograms, it is expected that the source of the anti‐tyrosinase activity of BSFC‐C is found within regions C001‐C007. Since the substances with [M‐H]^‐^ 339.1985 *m/z*, 325.1870 *m/z*, 311.1710 *m/z* are the major compounds in TSFC‐C and have very low anti‐tyrosinase activity, it is reasonable to assume that substances in C008‐C010 might hinder the enzyme inhibition.

C001–C007 areas contain more polar molecules, such as benzoic and phenylpropanoic acids, flavonoids, and glycosylated molecules, which might have tyrosinase inhibitors and antioxidant agents, as previously reported [20]. In the C003 the major contributor to the peak observed in both samples was, the ion 243.1230 *m/z* [M‐H]^‐^ (Figure 3). This pseudomolecular ion presents as fragments 225.1144, 207.1023, 181.1212, 163.1119 136.0527, 127.1114, 125.0969, and 110.0368 *m/z*. This fragmentation pattern was compatible with that observed for uridine [21], a natural nucleoside, consisting of a ribose sugar molecule linked to a uracil base. Tyrosinase inhibition is one of the bioactivities cited in the literature for uridine [22].

The main contributor to the C002 region was the ion [M‐H]^‐^ 163.0414 *m/z*, which was present in similar concentrations in both subfractions. This ion corresponds to *p*‐coumaric acid [15], the main precursor of flavonoid biosynthesis [5]. According to the literature, this compound has both antioxidant activity [20, 23] and anti‐tyrosinase activity [24].

The ion 225.1151 *m/z* [M‐H]^‐^, found in C005, was two times higher in BSFC‐C. In MS/MS, it presented the fragment ions at *m/z* 207.1024, 179.1062, 163.1127, 136.0521, 110.0371, and 97.0281. According to MS‐Finder, this substance could be porphobilinogen, a bioprecursor of porphyrins [25], which could be related to the low chlorophyll contents in this sample, which is a porphyrin derivative [25].

In C009, the ion 297.2460 *m/z* [M‐H]^‐^ was present in both samples but about 2.5 times higher in BSFC‐C. This compound presented fragment ions at *m/z* 297.2409, 279.2320, 261.2217, 171.1022, 155.1067, 141.12127.1111, 96.9594, and 79.9568 *m/z*. For this compound, MS‐Finder suggested the structure of an acyclic terpene with molecular formula C_18_H_34_O_3_ (298.2507 Da), with a m/z error of approximately 3 ppm, which agrees with an observed retention time of 45.17 min.

Subfraction D (Figure 4) presented the largest difference in sample composition than the others. Most of the BSFC‐D components were found in the C011 and C012 regions, while TSFC‐D compounds were found only inside the C013 area, with the main peak at 555.2865 [M‐H]^‐^ *m/z*, in a concentration approximately 11 times higher than in BSFC‐D. A summary of all annotaded compounds is presented in Table 4.

**TABLE 4:** List of annotated metabolites in Leaf extracts of *Guadua angustifolia* collected in Bauru and Tatuí—SP, Brazil.

Molecules or class	Chromatogram Region	[M‐H]^‐^ (*m/z*)	Annotation level [26]	MS^2^ (*m/z*)
*p*‐coumaric acid	C002, C011, C016	163.0414	2	119.0498; 93.0341
Uridine	C003	243.1230	2	225.1144; 207.1023; 181.1212; 163.1119; 136.0527; 127.1114; 125.0969; 110.0368
Porphobilinogen	C005	225.1151	2	207.1024; 179.1062; 163.1127; 136.0521; 110.0371; 97.0281
Cyclic fatty acid	C008	311.1710	3	311.1710; 183.0111
Acyclic terpene (C_18_H_34_O_3_)	C009	297.2460	3	297.2409; 279.2320; 261.2217; 171.1022; 155.1067; 141.1271; 127.1111; 96.9594; 79.9568
Cyclic fatty acids	C010, C013, C019, C020	311.1710	3	311.1710; 183.0111
		325.1870	3	325.1870; 183.0111
		339.2030	3	339.2030; 183.0111
Protocatechuic acid derivative	C011	431.1937	3	205.1234; 153.0911; 113.0233; 89.0243
Hydroxylated unsaturated long‐chain fatty acid (C_18_H_34_O_5_)	C012, C017	329.2321	3	229.1444; 211.1325; 193.1230; 183.1377; 171.1015; 139.1124; 127.1118; 99.0812
Not identified	C013, C019	555.2865	4	555.2865

The pseudomolecular ion 163.0393 *m/z* has already been discussed previously (C002) and is *p*‐coumaric acid, in this case, it is around 18 times more concentrated in BSFC‐D than in TSFC‐D. The concentration of p‐coumaric acid in this subfraction is even higher than in BSFC‐C.

The [M‐H]^‐^ 329.2321 *m/z* was found in BSFC‐D (C012) and showed the fragments at *m/z* 229.1444, 211.1325, 193.1230, 183.1377, 171.1015, 139.1124, 127.1118 and 99.0812. According to MS Finder, this [M‐H]^‐^ and fragmentation pattern indicates the possibility of a hydroxylated unsaturated long‐chain fatty acid with molecular formula C_18_H_34_O_5_ with *m/z* error 3.7 ppm, this class of molecules can present anti‐tyrosinase activity [27].

The pseudomolecular ion 431.1937 *m/z* (C011) was 12 times higher in BSFC‐D, showing MS [2] fragments at *m/z* 205.1234, 153.0911, 113.0233 and 89.0243. According to MS‐Finder, this compound can be a protocatechuic acid derivative, since it presents UV‐Vis absorbance indicating the presence of an aromatic ring and the characteristic ion at 153.0911 *m/z* [C_7_H_5_O_4_]^‐^. The loss of 226 *m/z* [[M‐H]‐205.1234] indicates possible glycoside esterification due to the high *m/z* loss value, which is a characteristic of glycosylated compounds. According to the literature, protocatechuic acid derivatives can express antioxidant and anti‐tyrosinase activity [28].

In the E subfractions, among the seven demarcated regions, five of them (C014, C015, C016, C017, and C019) are predominant in the Bauru specimen. Some of the compounds have already been previously commented on in this text and may contribute to the antioxidant and anti‐tyrosinase activity: *p*‐coumaric acid (C016) and 329.2322 *m/z* (C017), both being respectively 5 and 3 times more concentrated in this specimen. In the C019 region, it was also found a compound with [M‐H]^‐^ 555.2827 *m/z*, as reported for C013 which does not interfere with the activity tests, since the BSFC‐E concentration is as high as in TSFC‐D and its anti‐tyrosinase activity is still high. In the same region, the compound 325.1861 *m/z* [M‐H]^‐^, was also present in TSFC‐C (C010) and TSFC‐D (C013), which contributes to the hypothesis that they are contributing negatively to the measured activities.

The C020 region was the only one in which TSFD‐E presented compounds in a much higher concentration than the Bauru specimen. The major compound in this area was *m/z* 339.2021 [M‐H]‐, which was absent in BSFC‐E. This area also exhibited a pseudomolecular ion m/z 297.1549 [M‐H]‐ with a characteristic fragmentation pattern, including a fragment at m/z 183.0111. This fragmentation pattern, also observed in C010, strongly suggests that these compounds belong to the fatty acid class.

In summary, the analysis of the foliar extracts of bamboo from Bauru and Tatuí revealed significant differences in chemical composition and biological activities. The Bauru specimens showed higher antioxidant and tyrosinase inhibitory activity, which was attributed to the presence of phenolic compounds, benzoic and phenylpropanoic acids, fatty acids, and nucleosides, especially in BSFC‐C and BSFC‐D. Additionally, uridine was identified as a possible contributor to the anti‐tyrosinase activity in Bauru. On the other hand, the Tatuí specimens showed a higher concentration of fatty acids and terpenes, which did not show a significant impact on the evaluated activities.

The secondary metabolite may vary within the same species, mainly due to factors such as age, genetics, stress, and environmental conditions [29, 30]. Tatuí and Bauru, both situated in São Paulo state, exhibit significant differences in soil composition and climatic characteristics.

Tatuí’s terrain is predominantly composed of Latossolos (Oxisols) and Argissolos (Ultisols), which are deeply weathered, well‐drained soils with low natural fertility and elevated aluminum saturation in the subsoil. The region experiences a humid subtropical climate (Cwa classification), characterized by distinct summer and winter seasons [31].

Conversely, Bauru's region is predominantly underlain by the Bauru Sandstone Formation, resulting in soils with lower natural fertility. The climate in Bauru is comparatively warmer, with average annual temperatures ranging from 21°C to 25°C, and it features a pronounced dry season during the winter months (detailed comparison is given in Supporting information S2) [32].

The production of phenolic compounds in *G. angustifolia* is significantly influenced by the type of fertilizer applied [33]. Chemical fertilizers, such as diammonium phosphate (DAP), enhance the availability of nitrogen and phosphorus in the soil, which in turn boosts phenolic content through its effects on chlorophyll and plant metabolic pathways. Organic fertilizers, like humus, contribute to overall soil quality but may initially immobilize nitrogen, potentially limiting phenolic synthesis by affecting the availability of phenylalanine, a key precursor [33]. Among biological fertilizers, Promofort and Pseudomonas fluorescens stand out for their ability to stimulate the biosynthesis of phenolics and flavonoids through the release of indole derivatives. These biofertilizers enhance gene expression pathways critical to secondary metabolite production [33]. Additionally, they influence the rhizospheric microbial communities, indirectly promoting phenolic accumulation. This highlights the complex interaction between fertilization type, microbial activity, and plant metabolism, emphasizing the importance of tailored fertilization strategies to maximize secondary metabolite production in plants.

The location where the plant grows and the stresses to which it is subjected might influence its chemical composition; as well as water stress and herbivory can activate different metabolic pathways. For example, in *Vicia faba* L. plants under moderate water stress, the total emission of volatile compounds was significantly lower in infested plants than in non‐infested plants [34].

Environmental conditions, such as temperature, humidity, and nutrient availability, also influence the production of secondary metabolites. In a study with different tea cultivars, the accumulation of nerolidol glucoside was consistent with the expression level of UDP‐glucosyltransferase UGT91Q2 in response to cold stress [35].

The age of the plant also affects the composition of secondary metabolites. In a study with *Dendrocalamus giganteus* Munro, the lignin content increased with plant age, ranging from 22.8% to 29.7% in 1–4‐year‐old plants [36].

Understanding the influence of location and environmental factors on the manifestation of secondary metabolites is crucial to optimizing the use of plants for different purposes and understanding environmental interactions. Furthermore, these factors can help to comprehend biochemical pathways and survival mechanisms.

## 3 Conclusions

This study demonstrated the substantial impact of geographic origin on the chemical composition and biological activity of *G. angustifolia* var. *bicolor* extracts, as well as the potential adaptive mechanisms exhibited by this species. Further investigations into the precise environmental factors driving these variations—such as soil physicochemical properties, climatic conditions, and ecological interactions—would provide deeper insights into the adaptive strategies, functional properties, and metabolic pathways of this bamboo species. Since this study highlighted the impact of the environment in which a plant grows, understanding how these phenotype expression mechanisms work can be used to manipulate the production of these secondary metabolites, allowing their use in medicine, which requires quality control to ensure the effectiveness of its products.

## 4 Experimental

### 4.1 Collection of Plant Material

One of the samples was collected on April 11, 2017, at the Research and Development Unit (UPD) in Tatuí (SP, Brazil) linked to the São Paulo Agribusiness Technology Agency (APTA) of the Secretariat of Agriculture and Supply. The other was collected in the Nursery of the Bamboo Experimentation Laboratory of the São Paulo State University, Bauru Campus. The collected plant material was then identified by Dr. Tarcísio Filgueiras of the Botanical Institute of São Paulo (São Paulo, SP, Brazil). A control sample was deposited in the Herbarium of this same institution under the numbers Moreno 603 and Moreno 604.

### 4.2 Extraction and Column Chromatography

The extraction was performed with a Soxhlet apparatus with 70% ethyl alcohol, using 36 g of dried and chopped leaves, until the material was completely exhausted. Once the process was complete, the solvent was eliminated in a rotary evaporator under reduced pressure. The dry plant extract was fractionated by solid‐liquid extraction with four portions of 100 mL of each solvent of increasing polarity, *n*‐hexane, and dichloromethane, for 10 min on a magnetic stirrer. Subsequently, the solvents were eliminated under reduced pressure with a rotary evaporator, resulting in four different fractions. The chloroform fractions were fractionated by column chromatography, using silica gel as the stationary phase. For this purpose, 40 g of silica gel was placed in a glass column with a diameter of 4 cm to a height of 5 cm, occupying a volume of approximately 63 mL. The samples were applied after mixing with 1 g of silica for sample using 500 mg of the chloroform fraction. The elution was performed with three portions of 90 mL of each of the polarities, totaling 270 mL of the solvent mixture in each subfraction. The eluents used were Hexane:Chloroform (50%, v/v), Chloroform (100%), Chloroform:Methanol (95%, v/v), Chloroform:Methanol (90%, v/v) and Chloroform: Methanol (80%, v/v).

### 4.3 Anti‐tyrosinase Activity

To determine the anti‐tyrosinase activity, a colorimetric test was performed with the tyrosinase enzyme, using *L*‐Dopa. The fractions and subfractions were dissolved in different solutions containing 18% DMSO, 2% Tween 20, and 80% phosphate buffer pH 6.8 so that the sample concentration was between 0.9 and 1.1 mg/mL. The buffer was prepared by mixing 50 mL of a 0.027 g/mL K_2_HPO_4_ solution, 22.4 mL of a 0.8 mg/mL NaOH solution, and 127.6 mL of deionized water. *L*‐Dopa was dissolved in phosphate buffer to generate a 0.5 mg/mL solution to be used later during the procedure. Finally, the tyrosinase enzyme was prepared using 1 mg of the same and dissolving it in 10 mL of phosphate buffer (concentration of 0.1 mg/mL), and then a 1 mL aliquot was removed from this solution and 2 mL (contraction of 0.033 mg/mL) was added to perform the experiment. Different incubations were prepared in the 96‐well plate: containing 80 µL of buffer, 40 µL of the sample, and 40 µL of the tyrosinase solution; the sample blank, containing 120 µL of buffer and 40 µL of sample; the control with 80 µL of buffer, 40 µL of solvent (the same composition as the solution used to solubilize the sample) and 40 µL of the tyrosinase solution. The blank control contained 120 µL of buffer and 40 µL of solvent. Once the plate was prepared, it was left to incubate at 30°C for 5 min in a spectrophotometer. After this period, the plate was removed from the incubation and 40 µL of *L*‐Dopa was added to all wells. The plate was then incubated again at 30°C for 5 min, and the reading was then performed at 495 nm. All experiments were performed in triplicates and three replicates. Kojic acid was used as a positive control for this test.

### 4.4 Antioxidant Activity

The colorimetric test with DPPH was used to quantify the antioxidant potential. The tests were performed with spectrophotometers by measuring absorbance in 96‐well plates at 517 nm. To perform the test, a solution of 0.02% DPPH in methanol was prepared. The following were placed in different wells on the plate: a control containing 50 µL of methanol and 150 µL of DPPH; a blank control of 200 µL of pure methanol; 50 µL of sample with 150 µL of DPPH; the blank sample with 50 µL of sample and 150 µL of methanol. The reaction was left in a place without light interference for 30 min before reading the plate. All experiments were performed in triplicates and three replicates. Quercetin was used as the positive standard for this test. For IC_50_ values, analysis of variance statistical tests were performed.

### 4.5 HPLC‐MS/MS Conditions

The HPLC‐MS/MS was performed by the Analytical Center of the Chemistry Institute of the University of São Paulo, using the coupled equipment: qTof MAXIS 3GBruker Daltonics and HPLC Shimadzu LC 20AD XR. The chromatographic column used was Phenomenex Luna C18 5 µm (250 × 4.6 mm) at 40°C using the solvents H_2_O 0.1% AF (Solvent A) and Acetonitrile 0.1% AF (Solvent B), whose gradient was: 2% solvent B 0–2 min, gradient 2%–100% solvent B 2–60 min, 100% solvent B 60–65 min, gradient 100%–2% solvent B 65–66 min, 2% solvent B 66–70 min under the flow of 1.0 mL/min. While the mass spectrometry was performed in a system at 250°C and 2 Bar with an electrospray ionization source, and nine ion detectors in negative mode, where MS^1^ and MS^2^ were captured. In this case, negative mode detection was preferred due to the greater amount of information, both in databases and in the literature, on spectra of flavonoids and phenolic compounds.

### 4.6 Deconvolution

The chromatograms were deconvoluted using MZMine 4.2.0 software. The Mass Detection parameters were: MS1, level = 1, RT auto range, any polarity, any spectrum type, all scan types, factor of lowest signal, and noise factor 6.000. In the chromatogram builder, the retention time was adjusted according to the peaks to be treated with MS level = 1, the minimum number of consecutive scans was 8, the minimum intensity of consecutive scans was 2.0E2, the minimum absolute height was 8.0E3 and 5 ppm *m/z* tolerance (scan‐to‐scan).(Supporting information S1)

### 4.7 MS Finder

The MS‐Finder 3.61 software was used as a tool to guide the searches performed to find mass spectra in the literature that matched those observed. The following parameters were used to start the search: Fragmenter score ≥80; Database score ≥88; Substructure score ≥30.

## Author Contributions

All authors contributed equally to the manuscript preparation and design.

## Conflicts of Interest

The authors declare no conflicts of interest.

## Declaration of Generative AI and AI‐assisted Technologies in the Writing Process

During the preparation of this work, the authors used Co‐Pilot‐Microsoft to check the text's grammar and spelling. After using this tool/service, the authors reviewed and edited the content as needed and took full responsibility for the content of the publication.

## Supporting information

Supporting Information

Supporting Information

## Data Availability

Data are available on request from the authors.
