# Supplementary material for: Exploring the Discrepancies in the Biological Activities of Extracts From Guadua angustifolia Var. Bicolor Londoño Collected in Two Different Sites
Source: Chem Biodivers. 2025 May 3;22(9):e202500174. doi: 10.1002/cbdv.202500174 (PMC12435399; doi:10.1002/cbdv.202500174)
Supplement: Supplementary file 1 — Supporting Information [file CBDV-22-e202500174-s002.docx]

**Results**

Suplementary Tables

**Suplementary Table 1**- Results from the subfractions devonlution

| Reference | Tatui [M-H]^-^ (m/z) | Retention time (minutes) | Area Tatui | Bauru [M-H]^-^ (m/z) | Retention time (minutes) | Area Bauru |
| --- | --- | --- | --- | --- | --- | --- |
| C001 |  |  |  | 167.0341 | 12.63 | 4.6E3 |
|  | 225.1150 | 13.63 | 2.9E3 | 197.0446 | 13.06 | 1.3E4 |
|  | 197.0471 | 13.72 | 7.0E3 | 121.0291 | 13.56 | 3.9E4 |
| C002 | 173.0833 | 15.83 | 4.7E3 | - | - | - |
|  | 269.1417 | 15.85 | 1.5E4 | 269.1383 | 15.93 | 5.4E3 |
|  | 163.0414 | 15.96 | 2.7E4 | 163.0391 | 16.06 | 3.2E4 |
|  | 481.1749 | 16.40 | 5.8E3 | - | - | - |
| C003 | 187.0991 | 19.50 | 4.2E4 | 187.0966 | 19.46 | 1.1E4 |
|  |  |  |  | 243.1230 | 19.96 | 2.9E4 |
| C004 | 137.0256 | 20.67 | 2.7E3 | - |  |  |
|  | 229.1463 | 20.69 | 2.2E3 | 229.1435 | 20.70 | 3.3E3 |
|  | 257.1415 | 20.90 | 2.3E3 | - |  |  |
|  | 439.1902 | 20.97 | 5.4E3 | - |  |  |
|  | 433.1532 | 20.15 | 2.5E3 | 433.1486 | 20.17 | 1.3E4 |
|  | 243.1258 | 21.20 | 4.6E4 | 243.1230 | 19.96 | 2.9E4 |
| C005 | 453.2059 | 23.96 | 2.6E3 | 453.2008 | 23.97 | 9.6E3 |
|  | 225.1151 | 24.34 | 1.3E4 | 225.1123 | 24.31 | 2.6E4 |
| C006 | 551.1442 | 25.39 | 2.7E3 |  |  |  |
|  | 227.1307 | 25.40 | 4.8E3 | 227.1279 | 25.42 | 1.1E4 |
|  | 213.1149 | 25.47 | 4.0E3 |  |  |  |
|  | 215.1306 | 25.56 | 3.4E3 | 215.1280 | 25.84 | 6.3E3 |
|  | 327.2199 | 25.57 | 2.3E3 | 269.0446 | 26.01 | 3.5E3 |
|  | 417.1583 | 25.57 | 1.8E3 |  |  |  |
| C007 | 327.2199 | 28.29 | 1.6E4 | 329.2319 | 27.58 | 1.5E4 |
|  |  |  |  | 229.1437 | 28.73 | 2.3E3 |
| C008 | 307.1937 | 31.25 | 7.0E3 |  |  |  |
|  | 401.1895 | 31.44 | 1.6E3 | 401.1850 | 31.44 | 4.3E3 |
|  | 311.1887 | 32.35 | 1.2E4 | 311.1850 | 32.36 | 1.2E4 |
|  | 343.0847 | 33.41 | 9.8E3 | 343.0806 | 33.43 | 1.5E4 |
|  | 309.2094 | 34.30 | 1.8E4 | 309.2056 | 34.31 | 8.3E3 |
|  | 311.2248 | 34.52 | 1.4E4 |  |  |  |
|  | 343.2152 | 35.71 | 3.9E3 |  |  |  |
|  | 685.1968 | 35.93 | 5.5E3 | 685.1899 | 35.96 | 4.6E3 |
|  | 345.1865 | 36.29 | 4.3E3 |  |  |  |
|  | 667.1861 | 36.71 | 6.7E3 | 667.1794 | 36.63 | 6.1E3 |
|  | 313.2406 | 37.21 | 1.4E4 |  |  |  |
|  | 347.2020 | 38.33 | 5.3E3 | 347.1980 | 38.30 | 6.7E3 |
|  | 315.2564 | 39.86 | 1.1E4 | 315.2525 | 39.86 | 7.4E3 |
|  | 293.2144 | 40.01 | 4.1E3 |  |  |  |
|  | 713.2280 | 41.34 | 5.2E3 | 713.2209 | 41.39 | 4.9E3 |
|  | 295.2302 | 42.51 | 1.3E4 | 295.2265 | 42.31 | 1.0E4 |
|  | 243.1986 | 43.23 | 3.3E3 |  |  |  |
|  | 311.1710 | 43.65 | 1.8E5 |  |  |  |
| C009 |  |  |  | 595.4919 | 45.02 | 7.1E3 |
|  | 297.2460 | 45.03 | 2.2E4 | 297.2422 | 45.03 | 5.7E4 |
| C010 | 269.2148 | 46.49 | 1.3E4 | 269.2111 | 46.46 | 6.7E3 |
|  | 311.1710 | 46.58 | 3.5E4 |  |  |  |
|  |  |  |  | 327.2524 | 46.69 | 2.0E3 |
|  |  |  |  | 295.2264 | 46.76 | 6.9E3 |
|  |  |  |  | 341.2680 | 49.06 | 3.5E3 |
|  | 271.2303 | 49.22 | 6.0E3 | 271.2267 | 49.22 | 2.3E3 |
|  | 325.1870 | 51.82 | 3.2E5 | 325.1826 | 52.05 | 2.5E4 |
|  | 375.3149 | 53.33 | 5.7E3 | 375.3102 | 53.28 | 6.0E3 |
|  |  |  |  | 353.3045 | 53.66 | 3.2E3 |
|  |  |  |  | 369.2994 | 53.72 | 3.4E3 |
|  |  |  |  | 383.3150 | 55.84 | 2.5E3 |
|  | 555.2885 | 54.50 | 1.4E4 |  |  |  |
|  | 313.2773 | 55.71 | 1.0E4 | 313.2733 | 53.69 | 1.2E4 |
|  | 402.3043 | 56.26 | 3.2E3 |  |  |  |
|  | 299.2616 | 56.47 | 4.7E3 |  |  |  |
|  | 328.2965 | 56.69 | 6.2E3 |  |  |  |
|  | 327.2932 | 56.71 | 3.4E4 | 327.2891 | 56.65 | 3.8E4 |
|  | 255.2355 | 56.83 | 7.7E3 |  |  |  |
|  | 397.3355 | 57.85 | 1.8E3 | 397.3305 | 57.78 | 2.5E3 |
|  |  |  |  | 478.3517 | 57.97 | 2.5E3 |
|  |  |  |  | 339.1985 | 59.10 | 5.9E4 |
|  | 471.3515 | 59.17 | 3.8E3 | 471.3458 | 59.16 | 4.1E3 |
|  | 340.2060 | 59.79 | 1.3E5 |  |  |  |
|  | 341.2018 | 59.96 | 1.8E4 |  |  |  |
|  | 339.2030 | 60.14 | 6.8E5 |  |  |  |
|  | 355.3247 | 64.24 | 2.3E3 |  |  |  |
|  |  |  |  | 265.1469 | 59.60 | 4.2E3 |
| C011 |  |  |  | 584.2330 | 11.25 | 2.6E3 |
|  |  |  |  | 447.1857 | 11.28 | 6.9E3 |
|  |  |  |  | 182.0451 | 11.46 | 2.4E3 |
|  |  |  |  | 138.0555 | 11.47 | 7.7E3 |
|  | 137.0250 | 11.36 | 2.1E3 | 137.0240 | 12.06 | 4.7E4 |
|  |  |  |  | 487.1444 | 12.69 | 2.4E3 |
|  | 289.1669 | 12.01 | 5.8E2 | 289.1649 | 12.96 | 3.1E3 |
|  |  |  |  | 323.0760 | 13.21 | 3.5E3 |
|  |  |  |  | 553.2274 | 13.46 | 5.0E3 |
|  |  |  |  | 385.1849 | 13.61 | 3.0E3 |
|  | 431.1937 | 12.61 | 6.7E3 | 431.1911 | 13.61 | 8.5E4 |
|  |  |  |  | 583.2378 | 13.67 | 6.1E3 |
|  |  |  |  | 403.1597 | 14.06 | 7.9E3 |
|  |  |  |  | 479.2490 | 15.76 | 8.4E3 |
|  | 163.0405 | 14.38 | 6.0E3 | 163.0393 | 16.26 | 1.1E5 |
| C012 | 329.2342 | 27.52 | 1.5E4 | 329.2321 | 27.63 | 4.8E4 |
| C013 |  |  |  | 398.3264 | 50.50 | 1.9E3 |
|  |  |  |  | 565.3572 | 52.32 | 3.5E3 |
|  |  |  |  | 325.1828 | 52.49 | 2.8E4 |
|  |  |  |  | 576.3729 | 52.52 | 3.7E3 |
|  |  |  |  | 655.4399 | 52.55 | 2.3E3 |
|  |  |  |  | 623.4136 | 52.86 | 5.8E3 |
|  | 555.2865 | 54.78 | 5.0E5 | 555.2822 | 54.06 | 4.3E4 |
|  |  |  |  | 343.2836 | 55.40 | 3.7E3 |
|  |  |  |  | 313.2733 | 55.78 | 7.9E3 |
|  |  |  |  | 402.2994 | 56.30 | 3.5E3 |
|  |  |  |  | 327.2891 | 56.74 | 8.1E3 |
|  |  |  |  | 655.5856 | 56.74 | 1.8E3 |
|  |  |  |  | 255.2321 | 56.78 | 4.4E3 |
|  |  |  |  | 653.4241 | 57.13 | 6.2E3 |
| C014 | 179.0568 | 2.79 | 1.4E3 | 179.0553 | 2.80 | 3.9E3 |
|  | 165.0410 | 2.84 | 1.7E3 | 165.0397 | 2.87 | 4.7E3 |
|  |  |  |  | 215.0320 | 2.89 | 2.2E3 |
| C015 | 451.2198 | 10.87 | 2.3E3 | 451.2170 | 10.98 | 4.2E3 |
|  | 138.0566 | 11.00 | 3.2E3 | 138.0555 | 11.11 | 6.4E3 |
|  |  |  |  | 182.0451 | 11.11 | 1.7E3 |
|  | 137.0251 | 11.40 | 4.6E3 | 137.0239 | 11.61 | 1.4E4 |
| C016 | 163.0408 | 15.85 | 7.3E3 | 163.0394 | 16.01 | 4.0E4 |
| C017 | 329.2347 | 27.59 | 1.1E4 | 329.2322 | 27.55 | 3.2E4 |
| C018 | 699.3843 | 41.69 | 4.6E3 | 699.3787 | 41.70 | 6.4E3 |
| C019 |  |  |  | 326.2689 | 52.18 | 3.3E3 |
|  | 325.1861 | 53.52 | 7.4E4 |  |  |  |
|  |  |  |  | 555.2827 | 53.92 | 1.5E5 |
|  | 343.2872 | 55.32 | 4.0E3 | 343.2839 | 55.36 | 4.2E3 |
|  |  |  |  | 313.2735 | 55.73 | 4.7E3 |
|  |  |  |  | 402.2999 | 56.25 | 3.8E3 |
|  | 327.2924 | 56.56 | 2.7E3 | 327.2893 | 56.61 | 5.3E3 |
|  | 255.2347 | 56.73 | 2.5E3 | 255.2321 | 56.73 | 2.6E3 |
| C020 | 265.1500 | 59.83 | 3.8E4 | 265.1472 | 59.77 | 2.1E4 |
|  | 339.2021 | 60.13 | 1.9E5 |  |  |  |
|  | 327.2923 | 60.64 | 3.5E3 | 327.2891 | 60.64 | 2.2E3 |
|  | 297.1549 | 61.28 | 1.0E5 | 297.1517 | 60.83 | 1.1E4 |
|  | 309.1759 | 62.49 | 1.4E4 | 309.1727 | 61.94 | 8.6E3 |
|  | 279.1653 | 62.78 | 2.3E4 |  |  |  |
