# Supplementary material for: Exploring the Discrepancies in the Biological Activities of Extracts From Guadua angustifolia Var. Bicolor Londoño Collected in Two Different Sites
Source: Chem Biodivers. 2025 May 3;22(9):e202500174. doi: 10.1002/cbdv.202500174 (PMC12435399; doi:10.1002/cbdv.202500174)
Supplement: Supplementary file 2 — Supporting Information [file CBDV-22-e202500174-s001.docx]

**Suplementary material Table 2-** Comparison of Natural Conditions and Land Use in Bauru and Tatuí

|  | **Bauru** | **Tatuí** |
| --- | --- | --- |
| Climate | Dry winter mesothermal (Cwb) | Dry winter mesothermal (Cwa) |
| Average annual temperature | 20,2 °C | 20,2 °C |
| Average annual rainfalll | 1.258,6 mm | 1.260 mm |
| Relief | Wavy to strong wavy | Flat to wavy |
| Geology | Sandstone (Marília Formation) and basalt (Serra Geral Formation) | Siltstones and sandstones (Tatuí Formation) |
| Predominant soils | Red Nitosols and Red-Yellow Argisols | Nitosols and Latosols |
|  |  |  |
|  |  |  |
| Original vegetation | Semideciduous Seasonal Forest and Cerrado | Semideciduous Seasonal Forest and Cerrado |
| Current land use | Pasture, agriculture and reforestation | Agriculture and agronomic experimentation |
| Prevailing restrictions | Chemical (aluminum saturation) and physical (compaction) | Chemicals (aluminum saturation) |
